# Age-specific sex difference in the incidence of hepatocellular carcinoma in the United States

**DOI:** 10.18632/oncotarget.19245

**Published:** 2017-07-12

**Authors:** Pian Liu, Shao-Hua Xie, Shaobo Hu, Xiang Cheng, Tianyi Gao, Chen Zhang, Zifang Song

**Affiliations:** ^1^ Cancer Center, Union Hospital, Tongji Medical College, Huazhong University of Science and Technology, Wuhan 430022, China; ^2^ Department of Molecular Medicine and Surgery, Karolinska Institutet, Karolinska University Hospital, Stockholm 17176, Sweden; ^3^ Department of Hepatobiliary Surgery, Union Hospital, Tongji Medical College, Huazhong University of Science and Technology, Wuhan 430022, China; ^4^ The First Clinical Medical School, Tongji Medical College, Huazhong University of Science and Technology, Wuhan 430022, China

**Keywords:** hepatocellular carcinoma, liver cancer, sex difference, incidence, estrogen

## Abstract

**Background:**

Hepatocellular carcinoma possesses a notable sex difference in incidence, and a protective role of estrogens has been hypothesized.

**Methods:**

Using data from 13 cancer registries in the Surveillance, Epidemiology, and End Results Program, we describe the age-specific sex difference in the incidence of hepatocellular carcinoma in the United States during 1992-2013. We used a curve fitting by non-linear regression to quantitatively characterize the age-specific incidence rate of hepatocellular carcinoma by sex.

**Results:**

A total of 44,287 incident cases of hepatocellular carcinoma (33,196 males and 11,091 females) were included, with an overall male-to-female ratio in age-standardized rate of 3.55. The sex ratio was below 2 at ages <25 years, increased with age from ages 25-29 years until peaking at 5.40 at ages 50-54 years, and declined thereafter. We also observed additional peaks in the age-specific sex ratio curves at ages 25-34 years across racial/ethnic groups. Modelling of age-specific incidence rates indicated a 15-year delayed increase with age in females compared with males in Asian and Pacific Islanders, and an 11-year delay in Hispanic whites.

**Conclusions:**

The age-dependent patterns in the sex difference in the incidence of hepatocellular carcinoma support the hypothesis of a protective role of estrogens. The underlying reasons for the sex difference in hepatocellular carcinoma remain to be further explored in analytic epidemiological studies.

## INTRODUCTION

Hepatocellular carcinoma (HCC) is currently the third most common type of malignancy in men and seventh in women worldwide [1]. Major risk factors for HCC include chronic infection with hepatitis B virus (HBV) or hepatitis C virus (HCV), alcoholic liver disease, nonalcoholic fatty liver disease, and probably diabetes or obesity [2]. HCC is characterized by marked male predominance in incidence. The male-to-female incidence ratio of HCC varies between 2:1 and 4:1 across populations [3]. The excess risk of HCC in men is possibly explained by a higher prevalence of established risk factors, e.g., persistent HBV or HCV infection, alcohol use, and smoking in men than in women, and also intrinsic exposures with differential distribution between the sexes [2–4].

It has been speculated that sex hormones, such as estrogen, may be involved in the development of HCC [4–6]. However, existing epidemiological evidence remains sparse and conflicting. Based on data from the Surveillance, Epidemiology, and End Results (SEER) Program, we describe the age-specific sex difference in the incidence of HCC in the United States during the period 1992-2013. We hypothesized that a protective effect of estrogens against HCC development would be weaker after the menopausal ages due to decreased hormone levels in females, which would be reflected by a decline in the male-to-female ratio in incidence [7, 8]. We also assessed the age-specific sex difference in HCC incidence by race and calendar period to explore possible heterogeneities.

## RESULTS

A total of 44,287 incident cases of HCC (33,196 males and 11,091 females) were included in the analysis, including 5,590 cases (4,258 males and 1,332 females) in blacks, 10,731 (7,707 males and 3,024 females) in Asian and Pacific Islanders, 19,421 (14,889 males and 4,562 females) in non-Hispanic whites, 7,795 (5,814 males and 1,951 females) in Hispanic whites and 609 (415 males and 194 females) in American Indian/Alaska native. A total of 9,705 (22%) cases were diagnosed during the period 1992-1996, 13,554 (31%) during 1997-2006, and 21,055 (48%) from 2007 onwards. The overall male-to-female ratio in age-standardized incidence rate was 3.55 (95% confidence interval [CI]: 3.47, 3.62). The male-to-female ratio in HCC incidence was lower than 2 at ages below 25 years, increased with age from ages 25-29 years until peaking at ages 50-54 years (5.40, 95% CI: 5.02, 5.82), and showed a decline thereafter (Table 1).

**Table 1:** Age-specific incidence rate (1/100,000 population) of hepatocellular carcinoma by sex and male-to-female incidence ratios in the United States, 1992-2013

Age, years	Male		Female		Male-to-female ratio (95% confidence interval)
	Number	Rate	Number	Rate	
< 20	88	0.1	52	0.0	1.61 (1.14, 2.27)
20-24	50	0.2	35	0.1	1.36 (0.88, 2.09)
25-29	84	0.3	37	0.1	2.20 (1.50, 3.24)
30-34	159	0.5	52	0.2	2.99 (2.19, 4.09)
35-39	365	1.1	100	0.3	3.62 (2.90, 4.52)
40-44	853	2.6	183	0.6	4.70 (4.00, 5.51)
45-49	2,305	7.6	450	1.5	5.24 (4.74, 5.80)
50-54	4,318	16.2	832	3.0	5.40 (5.02, 5.82)
55-59	5,750	26.5	1,184	5.1	5.16 (4.84, 5.49)
60-64	5,280	30.9	1,344	7.2	4.31 (4.06, 4.57)
65-69	4,194	31.4	1,480	9.5	3.29 (3.10, 3.49)
70-74	3,735	35.4	1,703	12.9	2.74 (2.59, 2.90)
75-79	3,054	37.6	1,618	14.4	2.61 (2.46, 2.77)
80-84	1,847	34.0	1,191	13.7	2.47 (2.30, 2.66)
85+	1,114	27.7	830	9.5	2.93 (2.67, 3.20)
All	33,196	7.8	11,091	2.5	3.07 (3.01, 3.14)
Age-standardized rate *		8.7		2.4	3.55 (3.47, 3.62)

* Using 2000 United States Standard Population as reference.

**Figure 1:**
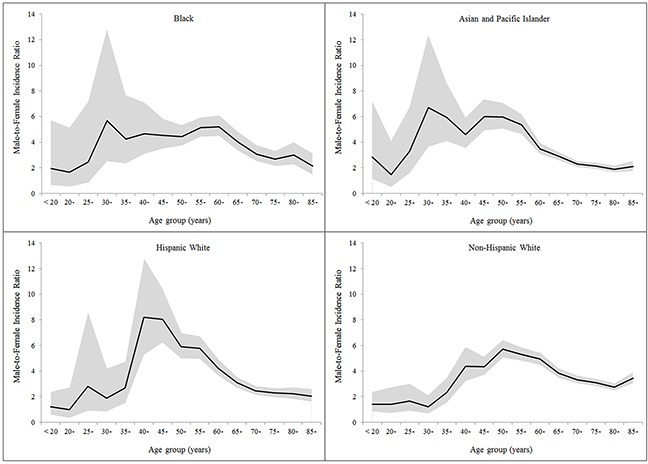
Age-specific male-to-female ratio in the incidence of hepatocellular carcinoma by racial/ethnic group

**Figure 2:**
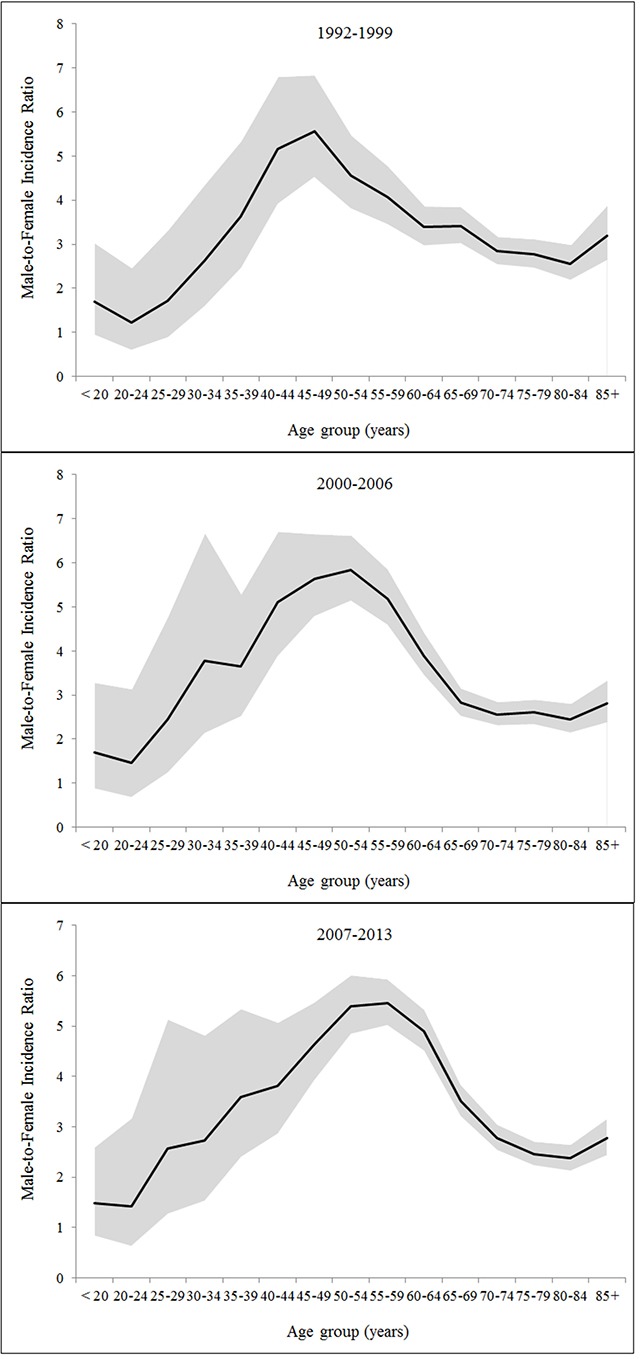
Age-specific male-to-female ratio in the incidence of hepatocellular carcinoma by calendar period

Despite the male-to-female ratio decreased after the ages 45-49 years or older in all racial/ethnic groups, it displayed distinct patterns across these groups. The ratio was as high as over 8 at ages of 40-49 years in Hispanic whites. The ratio was the highest at ages 30-34 years in blacks (5.68) and Asian and Pacific Islanders (6.72), which resulted in bimodal age-specific sex ratio curves. Minor peaks at ages 25-29 years were observed in the age-specific sex ratio curves in both Hispanic and non-Hispanic whites (Figure 1). Age-specific sex ratio curves displayed similar patterns across the three calendar periods (Figure 2).

**Figure 3:**
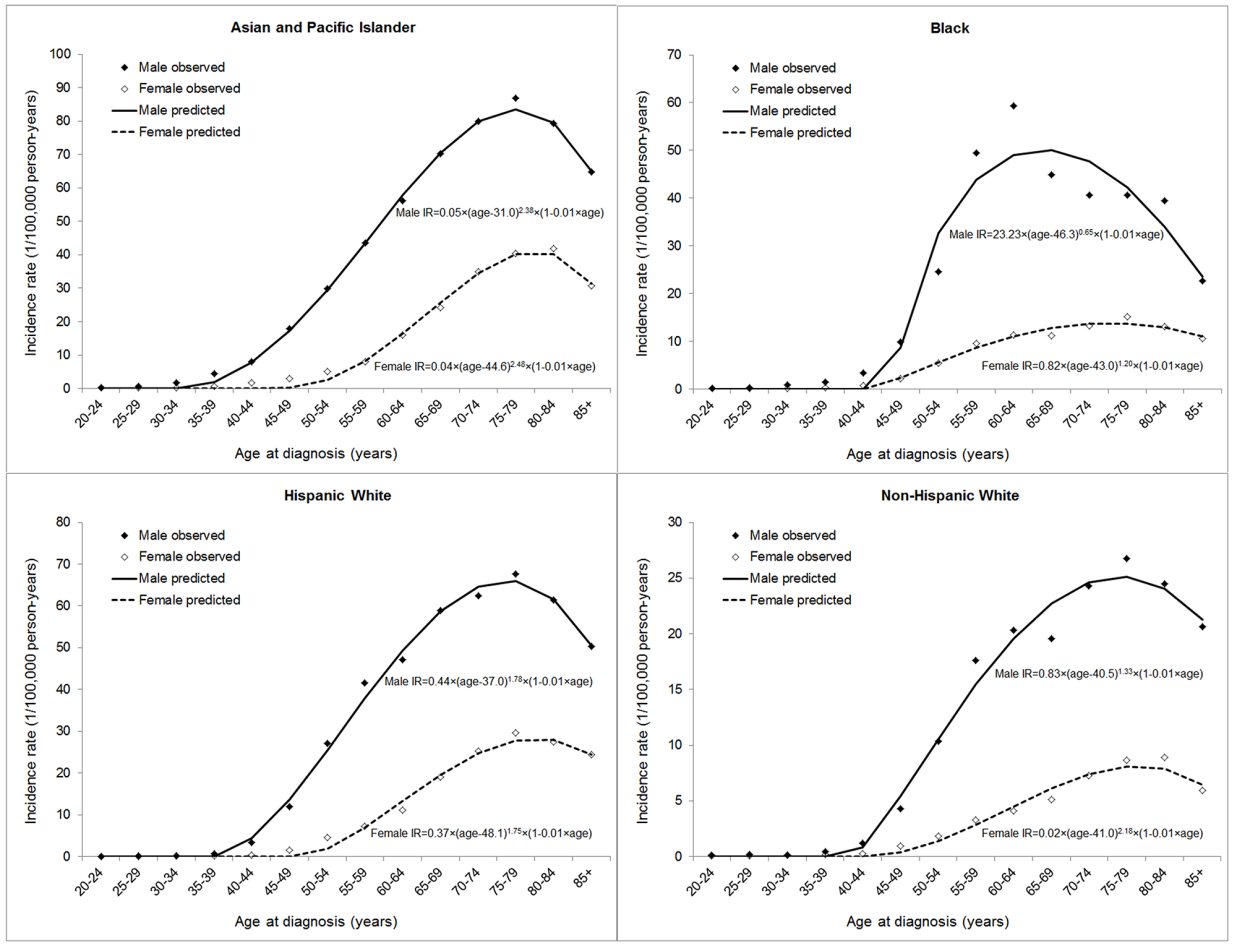
Modelling of age-specific incidence rate (IR) of hepatocellular carcinoma by sex and racial/ethnic group

Figure 3 shows the curve fitting of the age-specific incidence rates of HCC from non-linear regression by sex and racial/ethnic group. The ages at which the age-specific incidence curve increased above zero were similar between the sexes in blacks and non-Hispanic whites. However, the curves demonstrate divergent slopes between the sexes until the ages of 60-64 years. Modelling of the age-incidence curve showed a 15-year delayed increase with age in females (44.6 years) compared with males (31.0 years) in Asian and Pacific Islanders, and an 11-year delay in Hispanic whites (48.4 years in women versus 37.0 years in men).

## DISCUSSION

Using data from 13 cancer registries in the SEER program, we confirmed the male predominance in the incidence of HCC irrespective of race and ethnicity in the United States. Our findings showed the male-to-female ratio in HCC incidence increased with age until peaking at the ages 50-54 years and declined thereafter. We also observed additional peaks in the age-specific sex ratio curves at ages 25-34 years across racial/ethnic groups. Modelling of age-specific incidence rates indicated a 15-year delayed increase with age in females compared with males in Asian and Pacific Islanders, and an 11-year delay in Hispanic whites.

The decreased male-to-female ratio in HCC incidence since the menopausal ages and the delayed development of HCC in females in some racial/ethnic groups support our hypothesis of a protective effect of female sex hormones against HCC development. In addition, similar age-specific patterns in the sex difference of HCC incidence across calendar periods indicate that the sex difference in HCC incidence is more likely to be explained by intrinsic exposures or some stable environmental exposures, rather than extrinsic exposures with differential prevalence between the sexes which have changed over time. However, it is possibly related to some environmental exposures with differential prevalence between the sexes which have not changed over time, for example, heavy alcohol use [9]. Variations across racial/ethnic groups in the age-specific sex ratio curves, particularly for ages younger than 50 years, may be related to exposure in early life with differential distributions or effects on HCC risk between the sexes. Such exposures may include intrinsic and extrinsic exposures with sex hormonal effects, and childhood and adolescent obesity/overweight may be involved and warrants further investigations.

The reasons for the sex difference in HCC risk remain unclear. Although the prevalence of HBV infection has been modestly higher in men than in women in the United States [10], the male predominance of HCC remains in carriers of HBV [11]. On the contrary, the sex difference in HCV infection is minimal in the United States [10]. Despite the higher prevalence of tobacco smoking and heavy alcohol use in men than in women in the United States, the sex difference in the prevalence of smoking or heavy alcohol use does not substantially differ across age groups [9, 12], which does not correspond the age-dependent patterns in the sex difference of HCC incidence. Diabetes seems equally prevalent among men and women in most populations, or even showed a historical female excess in the first half of the last century [13], which is not in line with the male predominance in HCC. Overall, these main risk factors for HCC do not well explain the extreme male predominance in HCC incidence [14].

A protective role of female sex hormones and reproductive factors in the development of HCC has been hypothesized and examined in limited epidemio-logical studies. However, previous results have been conflicting and the statistical power has been limited by the low incidence of HCC in women [15–19]. Although the International Agency for Research on Cancer (IARC) has considered evidence that use of oral contraceptives increased risk of HCC as sufficient [20], a later meta-analysis has revealed that the association between oral contraceptives and HCC risk remained uncertain [21]. A recent population-based cohort study of 799,500 women in the United States found no association between parity, age at birth, age at natural menopause, duration of fertility, oral contraceptives use and HCC risk [18]. Overall, how sex hormonal and reproductive factors influence the risk HCC remain to be explored in large-scale investigations with sufficient statistical power.

Possible biological mechanisms underlying the possible protective role of estrogens may include the inhibition of inflammatory responses, prevention of oxidative stress, and inducing apoptotic cell death [4–6, 22]. It has been previously shown that the male predominance in HCC risk seems to present among chronic HBV carriers rather than HCV carriers [11], and HBV infection may pose higher HCC risk in men than in women [23]. Therefore, the male predominance of HCC is probably attributable to the interactions of HBV infection with the sex or other exposures related to the sex.

To the best of our knowledge, the present study is the first to characterize the age-specific sex difference in HCC incidence by racial/ethnic group. Incidence data of good quality in terms of case completeness and data accuracy have lent validity to our findings. However, some limitations of this study need to be discussed. First, this study was descriptive in nature where we used age as a proxy for sex hormone levels, particularly for a dramatic decrease of sex hormone levels in females after the menopausal ages. We were not able to evaluate the exact hormone levels and other sex hormonal exposures, including, but not restricted to, estrogens, androgens, oral contraceptives, and hormone replacement therapy. The hormonal hypothesis of HCC development remains to be tested in analytic epidemiological studies or controlled trials with information on sex hormonal exposures of individuals. The analysis was only based on data from the 13 cancer registries with expanded categories of race and ethnicity since 1992, and our findings may not be representative of the entire population of the United States or other populations. Due to the limited incidence of HCC, estimates of the sex ratios in incidence in some racial/ethnic groups or at early ages had relatively low precision, and need to be interpreted with caution. Finally, as information on the major risk factors of HCC is not available in the SEER database, we were unable to quantitatively examine how the sex difference in HCC incidence could be explained by gender disparities in these risk factors.

In summary, this SEER analysis displayed the age-dependent patterns in the sex difference in HCC incidence in the United States, which suggests a protective role of female sex hormones against HCC development. We also observed additional peaks in the age-specific sex ratio curves in early ages across racial/ethnic groups. More analytic epidemiological studies are still needed to clarify the underlying reasons for the sex difference in the risk of HCC.

## MATERIALS AND METHODS

### Data sources

The SEER Program of the National Cancer Institute is an authoritative source of information on cancer incidence and survival in the United States (seer.cancer.gov/seerstat/; version 8.3.2). We extracted incidence data on HCC (topography code C22 and histological codes 8170-8175 according to the International Classification of Diseases for Oncology, 3rd edition [ICD-O-3]) from the November 2015 submission of the SEER 13 registries database. The SEER 13 database includes all incident cases of HCC from 13 cancer registries (Atlanta, Connecticut, Detroit, Hawaii, Iowa, New Mexico, San Francisco-Oakland, Seattle-Puget Sound, Utah, Los Angeles, San Jose-Monterey, Rural Georgia and the Alaska Native Tumor Registry), which covers approximately 13.4% of the total population in the United States, since 1992 with expanded race categorization. Population estimates were from the SEER program, which uses a slight modification of the annual population estimates from the Census Bureau.

### Statistical analysis

We divided all cases into five-year groups beginning from ages 20-24 years, and all cases aged below 20 years or ≥85 years were combined into separate groups. We calculated the incidence rates by sex for each age group by dividing the number of cases by the corresponding population size. Age-standardized incidence rates by sex were calculated using the direct method with the 2000 United States Standard Population as reference. The male-to-female incidence ratios were calculated by dividing the rate in males by that in females for each age group, and the corresponding 95% CI was estimated based on the assumption of log-normal distribution. We further performed stratified analysis for the four major racial/ethnic groups (Non-Hispanic whites, Hispanic whites, blacks, and Asian and Pacific Islanders) and for the three approximately equally divided calendar periods 1992-1999, 2000-2006, and 2007-2013.

We further used a non-linear regression curve fitting approach to quantitatively characterize the age-specific incidence rates of HCC by sex. We fitted the equation *I*_(*t*)_ = *a* × (*t* − *d*)*^b^* × (1 − *kt*) to the age-specific incidence data using the SOLVER function of Microsoft Excel [7, 8, 24]. In this equation, *I*_(*t*)_ is the age-specific incidence rate of HCC at the age *t* (the mean age of the group), *a* is a scaling factor, *b* is a power term reflecting the rate of incidence change with age, *d* is a delayed term for the time between birth and age of increased incidence above zero, and *k* is an empirical term to adjust for decreasing rate at the oldest ages. A logic “if” function was used such that when *t* < *d*, *I*_(*t*)_ = 0. Thus, only when *d* > *t* was *I*_(*t*)_ > 0.
